# Ginsenoside Compound K Induces Ros-Mediated Apoptosis and Autophagic Inhibition in Human Neuroblastoma Cells In Vitro and In Vivo

**DOI:** 10.3390/ijms20174279

**Published:** 2019-09-01

**Authors:** Jung-Mi Oh, Eunhee Kim, Sungkun Chun

**Affiliations:** 1 Department of Physiology, Chonbuk National University Medical School, Jeonju 54907, Korea; 2 School of Life Sciences, Ulsan National Institute of Science and Technology, Ulsan 44919, Korea

**Keywords:** autophagy, apoptosis, ginsenoside, mitochondrial ROS, neuroblastoma

## Abstract

Autophagy can result in cellular adaptation, as well as cell survival or cell death. Modulation of autophagy is increasingly regarded as a promising cancer therapeutic approach. Ginsenoside compound K (CK), an active metabolite of ginsenosides isolated from *Panax ginseng* C.A. Meyer, has been identified to inhibit growth of cancer cell lines. However, the molecular mechanisms of CK effects on autophagy and neuroblastoma cell death have not yet been investigated. In the present study, CK inhibited neuroblastoma cell proliferation in vitro and in vivo. Treatment by CK also induced the accumulation of sub-G1 population, and caspase-dependent apoptosis in neuroblastoma cells. In addition, CK promotes autophagosome accumulation by inducing early-stage autophagy but inhibits autophagic flux by blocking of autophagosome and lysosome fusion, the step of late-stage autophagy. This effect of CK appears to be mediated through the induction of intracellular reactive oxygen species (ROS) and mitochondria membrane potential loss. Moreover, chloroquine, an autophagy flux inhibitor, further promoted CK-induced apoptosis, mitochondrial ROS induction, and mitochondria damage. Interestingly, those promoted phenomena were rescued by co-treatment with a ROS scavenging agent and an autophagy inducer. Taken together, our findings suggest that ginsenoside CK induced ROS-mediated apoptosis and autophagic flux inhibition, and the combination of CK with chloroquine, a pharmacological inhibitor of autophagy, may be a novel therapeutic potential for the treatment of neuroblastoma.

## 1. Introduction

Neuroblastoma is the second most prominent type of solid tumor in childhood, with more than 50% of cases occurring within two years of birth [1,2]. Despite advances in treatment modalities such as surgery, radiation therapy, and chemotherapy, neuroblastoma is generally progressive and very malignant, with long-term survival rates of <40% for children with advanced and metastatic disease [3,4,5]. Presently, the development of alternative medicines derived from plants with low side effects is being studied extensively [6]. Accumulating evidence reveals that these anticancer drugs induce various cell death mechanisms, including apoptosis and autophagy in several types of cancer cells [7,8,9]. Autophagy and apoptosis play an important role in determining cancer cell fate and are essential metabolic pathways for organism homeostasis, organ development, and cancer [8]. Apoptosis is a selective physiological process in which activation of caspases (cysteine aspartyl proteases) results in mitochondrial membrane permeability and morphological changes, including chromatin condensation and DNA fragmentation, thereby leading to programmed cell death [10]. Failure of apoptosis is thought to contribute to tumorigenesis, because it regulates the balance between cell proliferation and death. Therefore, targeting of key apoptotic modulators has become a strategy for the development of cancer therapies [11,12].

Similarly, autophagy is an evolutionarily conserved catabolic process of lysosomal degradation of cytoplasmic content in eukaryotes [12]. This autophagic pathway includes vesicle elongation, autophagosome maturation (sequestration of cargo), autophagosome–lysosome fusion, and degradation [13]. It provides energy for cell functions through degradation of molecules and cellular organs but also reduces cell damage by promoting the elimination of pathogens, toxic molecules, damaged cellular organelles, and misfolded proteins. However, excess autophagy can induce autophagic cell death by excessive degradation of mitochondria and damaged molecules required for cell survival [14,15,16]. Autophagy acts like a “double-edged sword” that plays a positive or negative role in cancer cells during the tumorigenesis process. Whether autophagy increases or inhibits cell death in response to cellular stress remains questionable [17]. Therefore, to balance cell survival and death, it is necessary to understand the complexity of the relationship between cancer cell apoptosis and autophagy.

**Figure 1:**
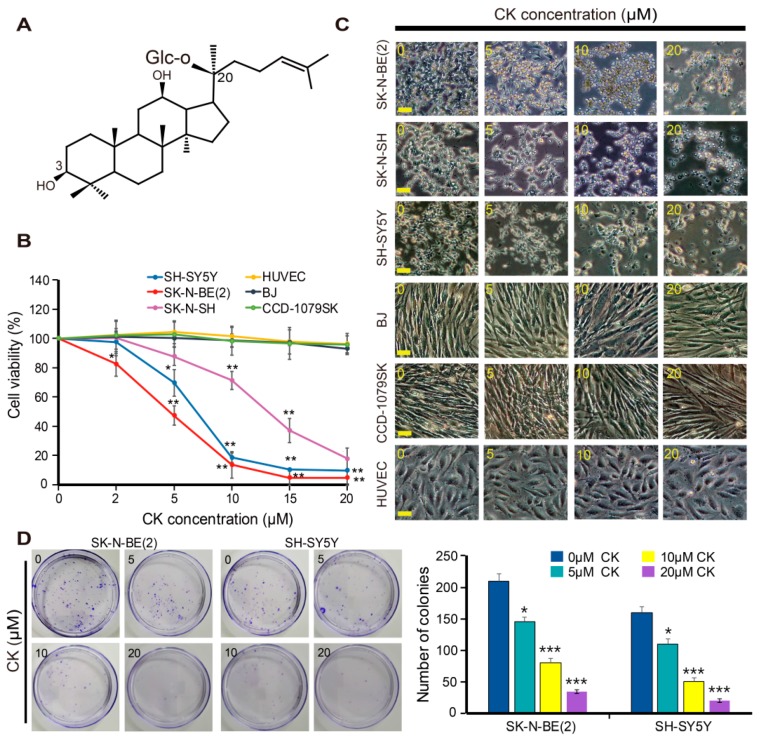
CK induces cell death in neuroblastoma cells. (**A**) Chemical structure of CK. (**B,C**) Three different neuroblastoma cells and normal cells were treated in various concentrations (0, 2, 5, 10, 15, and 20 µM) with CK, and cell viability was determined by CCK-8 assay. Data are presented as the mean ± SD of three independent experiments. *: *p* < 0.05 or **: *p* < 0.01 versus control. (**C**) Cell morphology change induced by CK treatment and cell morphology were observed under a microscope. Scale bar: 50 μm. (**D**) Representative images of colony formation assay in SK-N-BE(2) and SH-SY5Y. Data are presented as the mean ± SD of three independent experiments. *: *p* < 0.05 or ***: *p* < 0.001 compared to control. CK, Ginsenoside compound K.

*Panax ginseng* C.A. Meyer has been used as a health product and natural remedy in traditional medicine in many Asian countries such as China, Korea, and Japan for thousands of years [18]. Ginsenoside (ginseng saponins) is the major active component of ginseng, and more than 20 ginsenosides have been reported to possess various biological activities, including anti-inflammation, anti-carcinogenesis, anti-metastasis, and neuroprotection [19,20,21,22]. Compound K (CK) is a major metabolite component of several protopanaxadiol type (PPD) ginsenosides (Rb1, Rb2, and Rc) that is secreted by intestinal bacteria in humans and rats through the multistage cleavage of sugar moieties [23]. CK is a derivative of the PPD-ginsenoside, and its chemical formula is C_36_H_62_O_8_ with a molecular weight of 622.86 g/mol (Figure 1A). The biological function of CK has been explored for its antitumor and anti-inflammatory effects in several disease models [18,24,25]. CK blocks proliferation and migration of tumor cells and promotes apoptosis and autophagy [26,27,28]. However, its mechanism of action in neuroblastoma cells is unknown. Therefore, in the present study, we aimed to investigate the anticancer effects of CK and its underlying mechanisms on crosstalk between apoptosis and autophagy in neuroblastoma cell lines.

## 2. Results

### 2.1. CK Inhibits the Growth of Human Neuroblastoma Cells

To investigate the effect of CK on the growth of human neuroblastoma cells, three neuroblastoma cell lines, SK-N-BE(2), SH-SY5Y, and SK-N-SH cells, were cultured in the presence of various concentrations of CK (0–20 μM) for 24 h, and the cell viability was then assessed using a CCK-8 assay. A 24 h CK treatment significantly inhibited the growth of three neuroblastoma cell lines in a dose-dependent manner, with IC_50_ values of 5 μM [SK-N-BE(2)], 7 μM (SH-SY5Y), and 15 μM (SK-N-SH) cells, respectively (Figure 1B). SK-N-BE(2) and SH-SY5Y cells were more sensitive to CK than SK-N-SH cells, so these two cells were used for subsequent studies. Alternatively, CK showed no obvious anti-growth effects on CCD-1079SK, BJ, and HUVEC, as models of normal cells (Figure 1B). Following CK treatment, morphological changes of cells were observed by phase-contrast microscopy. Morphological changes included cell shrinkage, increased cell floating, and reduced cell attachment compared to untreated control cells (Figure 1C). To further confirm the inhibitory effect of CK on the proliferation of SK-N-BE(2) and SH-SY5Y cells, a colony formation assay was performed. As a result, the number of colonies was decreased in a dose-dependent manner after treatment with CK in both SK-N-BE(2) and SH-SY5Y cells (Figure 1D). Altogether, these results suggest that CK can inhibit neuroblastoma cell proliferation without affecting normal cells.

### 2.2. CK Induces Cell Cycle Arrest and Apoptotic Cell Death in Neuroblastoma Cells

**Figure 2:**
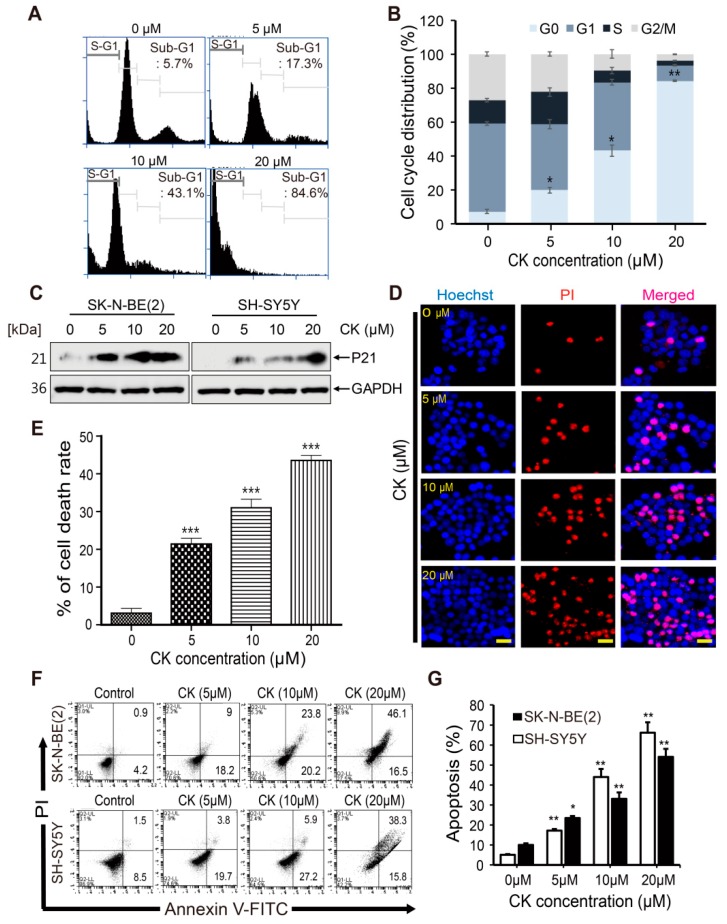
CK induced cell cycle arrest and apoptotic cell death in neuroblastoma cells. (**A**) Cell cycle distribution of SK-N-BE(2) cells treated with 0, 5, 10, and 20 µM CK for 24 h was detected and analyzed by flow cytometry following staining with PI. Quantitative analysis of cells in each cell cycle phase was performed. (**B**) Representative images of flow cytometry are shown. The experiment was repeated in triplicate. Data represent mean ± SD. *: *p* < 0.05 or **: *p* < 0.01 versus control. (**C**) The expression of P21 protein levels was examined by western blot after CK treatment for 24 h in neuroblastoma cells. (**D**) Nuclear structures of SK-N-BE(2) cells were analyzed via fluorescence microscope. The cells were fixed and stained with Hoechst 33342/PI solution for 10 min at room temperature. (**E**) The ratio of PI staining (red) and Hoechst 33342 staining (blue) represented cell death rates. Data are presented as the mean ± SD of three independent experiments. ***: *p* < 0.001 versus control. Scale bar: 50 μm. (**F**) Apoptotic cell death was determined by Annexin V/PI flow cytometry analysis. SK-N-BE(2) and SH-SY5Y cells were treated with different concentrations of CK (0, 5, 10, 20 μM) for 24 h. The results shown are representative of three independent cytometric analyses. Histograms illustrate the number of cells undergoing apoptosis. (**G**) The percentage of apoptotic cells is shown. Data are mean ± SD of three independent experiments. *: *p* < 0.05, **: *p* < 0.01 versus control group.

To determine the underlying mechanisms by which CK exerts cytotoxicity, we examined the cell cycle distribution in SK-N-BE (2) cells. SK-N-BE(2) cells were treated with various concentrations of CK for 24 h and then flow cytometry was performed. The results showed that CK significantly induced accumulation of the sub-G1population (apoptotic cells) in a dose-dependent manner (Figure 2A,B). Moreover, CK treatment increased the level of P21 protein, a potent inhibitor of cell cycle progression in SK-N-BE(2) and SH-SY5Y cells (Figure 2C). These results suggest that the CK-inhibited cell proliferation was due to cell cycle arrest at the sub-G1 phase in neuroblastoma cells.

To confirm the cell death in CK-treated SK-N-BE(2), we performed Hoechst/PI staining. For Hoechst/PI staining, SK-N-BE(2) cells were treated with various concentrations of CK for 24 h, and morphological changes of cells were observed by fluorescence microscopy. Hoechst 33342 dye is a membrane-permeable dye but PI is not. As membrane permeability increases in necrosis or later stages of apoptosis, PI forms a bright red, fluorescent complex across cell membranes. All nucleated cells can be stained blue with Hoechst 33342. Condensed and fragmented apoptotic cells stain brighter than normal nuclei do, so that apoptotic cells can be distinguished from living cells [10]. Cell nuclei emitting a fluorescence signal from both Hoechst and PI (fluorescence co-localization) were considered to be dead cells, while cells emitting only Hoechst signal were counted as live cells (Figure 2D). Cell death was quantitated by four visual fields randomly selected for each sample and the results were expressed as the ratio of PI positive cells to total cells (Hoechst positive cells). CK treatment in SK-N-BE(2) cells caused 20.5%, 31.3%, and 43.6% cell death at 5, 10, and 20 μM, respectively (Figure 2E). Next, we performed the flow cytometry using Annexin V-FITC/PI double staining. As shown in Figure 2F,G, CK significantly increased apoptotic cells in a dose-dependent manner in SK-N-BE(2) and SH-SY5Y cells. These findings suggest that CK can induce apoptotic cell death in neuroblastoma cells.

### 2.3. CK Induces Apoptotic Cell Death in Neuroblastoma Cells via Caspase Dependent- Pathways

**Figure 3:**
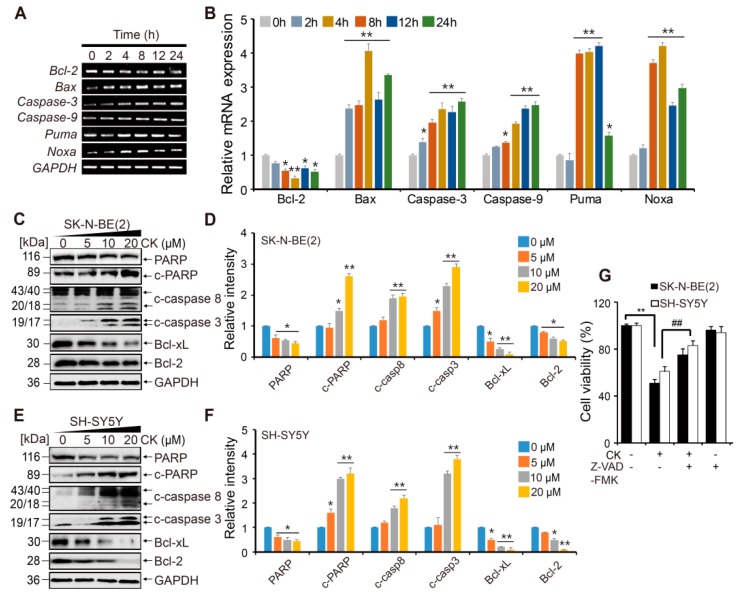
CK induces apoptotic cell death of human neuroblastoma cells. (**A**) mRNA expression levels of apoptotic genes, *Bcl-2*, *Bax*, *Caspase-3*, *Caspase-9*, *PUMA*, and *Noxa*, were evaluated by semi-quantitative reverse transcription polymerase chain reaction. (**B**) Data were obtained from at least three independent experiments and values are presented as the mean ± SD. *: *p* < 0.05, **: *p* < 0.01 versus control group. (**C**–**F**) Quantification of the protein levels were tested by western blot in SK-N-BE(2) and SH-SY5Y cells. Glyceraldehyde 3-phosphate dehydrogenase (GAPDH) was used as an RNA and protein loading control. Band intensities were quantitatively analyzed using the Image J program, and bar graphs in (**D**) and (**F**) are represented as mean ± SD of three independent experiments. *: *p* < 0.05, **: *p* < 0.01 versus control group. CK, Ginsenoside compound K. (**G**) Neuroblastoma cells were pretreated with or without 20 µM Z-VAD-FMK for 3 h and then treated with or without CK for 24 h. Cell viability was analyzed using the CCK-8 assay. Data error bars correspond to the mean ± SD of three independent experiments. **: *p* < 0.01 versus the control and ##: *p* < 0.01 versus CK-treated cells.

To explore the molecular mechanism of CK-induced apoptosis, we performed reverse transcription polymerase chain reaction (RT-PCR) and Western blots for apoptosis-related proteins. The expression of apoptosis-related genes (*Bcl-2*, *Bax*, *Caspase-3*, *Caspase-9*, *PUMA*, and *Noxa*) was measured in SK-N-BE(2) cells after 5 µM CK treatment for the indicated time. The results revealed that CK upregulated pro-apoptotic genes, including *Caspase-9*, *Caspase-3*, *Bax*, *Noxa*, and *PUMA*, in a time-dependent manner (Figure 3A,B). Meanwhile, CK significantly decreased the expression of anti-apoptotic gene *Bcl-2* in SK-N-BE(2) cells. In western blots, the protein level of cleaved caspase-3, caspase-8, and poly(ADP-ribose) polymerase (PARP) was significantly upregulated in SK-N-BE(2) and SH-SY5Y cells in a dose-dependent manner (Figure 3C–F). The Bcl-2 family proteins are known to be involved in the mitochondrial-mediated apoptosis pathway that activates the caspase cascade. Our results show that Bcl-2 family protein expressions, Bcl-2 and Bcl-xL (anti-apoptotic proteins), were downregulated (Figure 3C–F). These results indicate that CK strongly induces apoptosis in SK-N-BE(2) and SH-SY5Y cells by triggering caspase activation.

To elucidate further how CK induces NB cell death by apoptosis via caspase activation, we used a pan caspase inhibitor, Z-VAD-FMK. CCK-8 analysis showed that pretreatment with Z-VAD-FMK blocked cytotoxic effects induced by CK treatment in neuroblastoma cells (Figure 3G). This result suggests that CK induces caspase-dependent apoptosis in neuroblastoma cells.

### 2.4. CK-induced Apoptosis Involves ROS Generation in Neuroblastoma Cells

**Figure 4:**
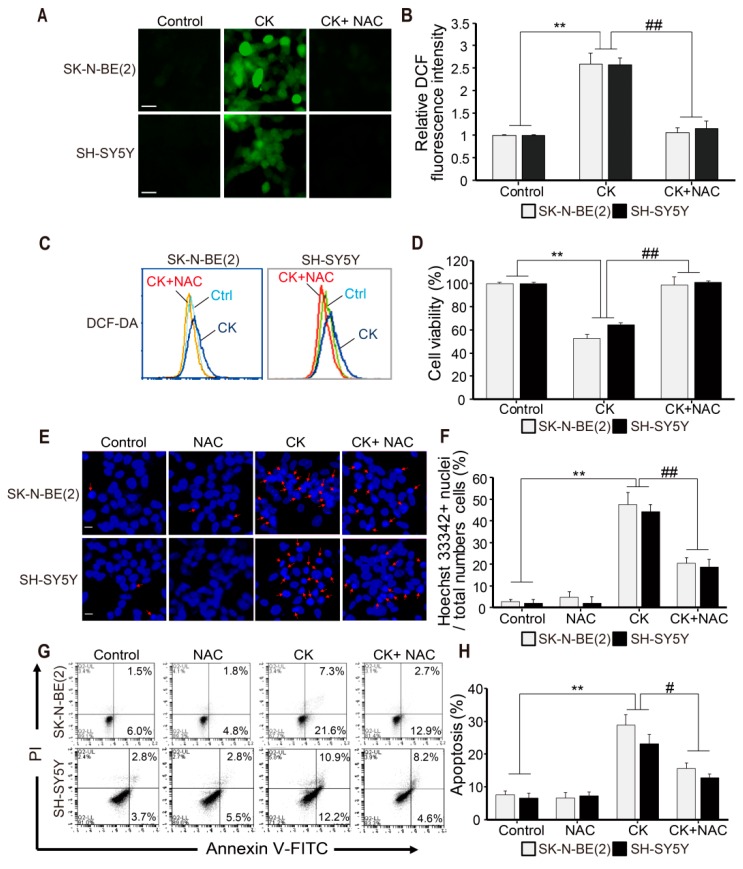
CK-induced apoptosis is associated with reactive oxygen species (ROS) generation in human neuroblastoma cells. (**A**–**H**) SK-N-BE (2) and SH-SY5Y cells were pretreated with 5 mM NAC for 3 h and treated with 5 µM CK for 24 h. (**A**) ROS generation that occurred in CK-treated cells was measured by fluorescence microscopy using DCFH-DA staining, and fluorescence intensity was shown as a histogram using the Image J program. (**B**) Relative DCF fluorescence intensities were expressed as fold of DCF fluorescence over the control in three groups (Control, CK, and CK + NAC). The effect of NAC on CK-induced ROS generation was also measured by flow cytometry (**C**), and the percentage of cell viability with CCK-8 assay was shown in the histogram (**D**). Data in B and D were shown as mean ± SD. **: *p* < 0.01 versus control and ##: *p* < 0.01 versus CK-treated alone. (**E**) After cells were stained with Hoechst 33342 dye, apoptotic bodies and chromatin-condensed nuclei (indicated by arrows) were observed under a fluorescence microscope. Scale bar: 20 μm. (**F**) The bars indicate the percentage of cells with apoptotic morphology among the total Hoechst 33342 stained cells. (**G**) Cells were stained with Annexin V/PI assay by flow cytometry. (**H**) The percentages of apoptotic cells (Annexin V + cells) are shown. All data (**F** and **H**) are shown as mean ± SD. **: *p* < 0.01 versus control and ##: *p* < 0.01 versus CK-treated alone.

Intracellular reactive oxygen species (ROS) production by mitochondria has been reported to be closely related to apoptosis in various cells [29,30]. Therefore, we investigated whether intracellular ROS activation was involved in CK-induced apoptosis. SK-N-BE(2) and SH-SY5Y cells were pretreated with or without 5 mM NAC for 3 h and then treated with CK for 24 h. ROS measurement with DCF-DA dyes was done by fluorescence microscopy and flow cytometry (Figure 4A–C). We found that CK treatment significantly enhanced intracellular ROS levels compared to untreated controls (Figure 4A–C). As expected, the ROS scavenger NAC markedly blocked CK-induced ROS generation in both SH-SY5Y and SK-N-BE(2) cells (Figure 4A–C). Through the CCK-8 assay, NAC pretreatment showed a significant recovery of CK-induced cell death (Figure 4D), whereas NAC treatment alone did not alter ROS levels and had no toxic effect on neuroblastoma cell viability (data not shown). In addition, we examined the effect of CK, NAC, or CK combined with NAC on apoptosis by Hoechst 33342 and Annexin V/PI staining (Figure 4E–H). As shown in Figure 4E,F, CK treatment significantly increased chromatin condensation (light blue staining cells) in neuroblastoma cells, while NAC treatment significantly inhibited chromatin condensation. In addition, CK significantly reduced Annexin V-positive cells in the presence of NAC (Figure 4G,H). Collectively, these results suggest that ROS generation is involved in CK-induced apoptosis of human neuroblastoma cells.

### 2.5. CK Induces Early-Stage Autophagy and Autophagosome Accumulation in Human Neuroblastoma Cells

**Figure 5:**
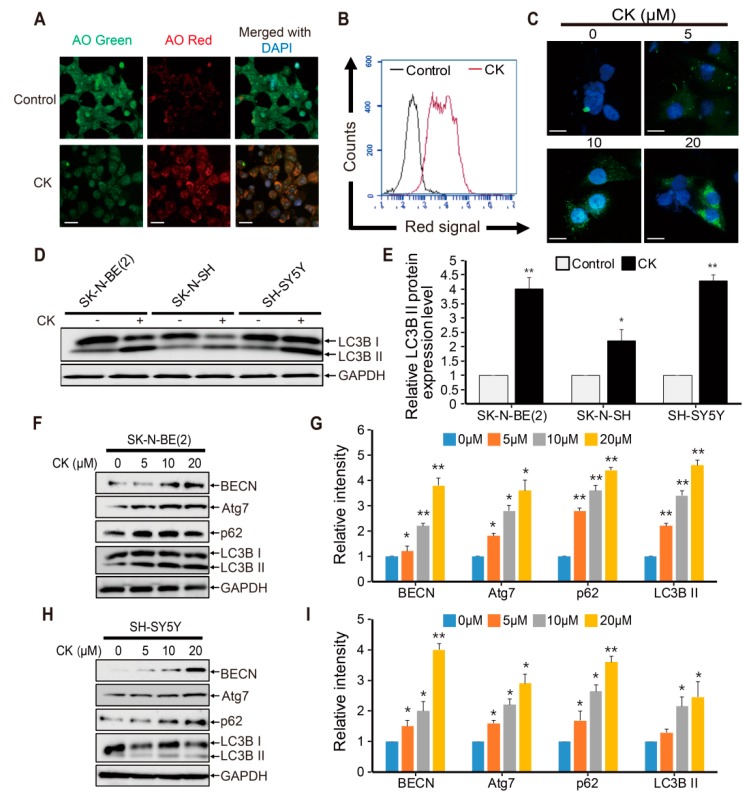
CK treatment promotes early stage autophagy but blocks autophagic flux. (**A**) Acidic vesicular organelles (AVOs) were examined by incubating SK-N-BE(2) cells treated with or without CK in 10 μM acridine orange (AO) for 30 min, and images were captured using a fluorescence microscopy. (**B**) AVO-positive SK-N-BE(2) cells were quantified by flow cytometry. Results shown are representative of three independent experiments. (**C**) pEGFP-LC3B transfected stable SK-N-BE(2) cells were treated with different concentrations of CK for 12 h, and images showing LC3-GFP puncta and accumulation of autophagosomes were visualized under a florescence microscope. CK treatment increases GFP-LC3 punctuation. Scale bar: 50 μm. (**D**) The LC3B expression level was measured by western blot in SK-N-BE(2), SK-N-SH, and SH-SY5Y cells treated with 5 μM of CK for 24 h. (**E**) The relative LC3B II protein expression level was significantly increased by CK treatment in all three neuroblastoma cell lines. (**F**–**I**) Cells were treated with various concentrations of CK for 24h in SK-N-BE(2) and SH-SY5Y cells. Western blotting was performed with antibodies specific for BECN, Atg7, p62, LC3B, and GAPDH in SK-N-BE(2) cells (**F**) and SH-SY5Y cells (**H**). Relative protein expression levels were shown in the histogram (**G,I**), and band intensities were quantified using the Image J program. All bar graph data (**E**,**G**,**I**) are shown as mean ± SD in three independent experiments. *: *p* < 0.05 versus control and **: *p* < 0.01 versus control.

The crosstalk between autophagy and apoptotic cell death was previously reported in various cancer cell types [10,11,12,13,14]. Hence, to investigate whether CK can act as an autophagy modulator, acridine orange (AO) staining was performed in CK-treated SK-N-BE(2) cells. Because autophagy-related cell death is characterized by the accumulation of vesicles and the formation of autophagosomes, AO staining has been used to analyze the formation of acidic vesicle organelles (AVOs) or autolysosome vacuoles, resulting from the fusion between autophagosomes and lysosomes, which is a key feature of autophagy [31]. In this study, CK not only significantly enhanced the accumulation of autophagic vacuoles (red fluorescence) in SK-N-BE(2) cells (Figure 5A), but flow cytometry verified that the proportion of AO-positive cells was increased after CK treatment (Figure 5B). The microtubule-associated protein light chain (LC)3 was used to monitor autophagy by detecting the switch from LC3BI to LC3BII, and GFP-LC3 puncta was used for transfecting mammalian cells to study autophagy [32].

For that, SK-N-BE(2) cells stably expressing GFP-LC3 were seeded, treated with increasing concentrations of CK for 24 h, and observed using fluorescence microscopy. CK treatment markedly increased GFP-LC3 puncta formation in cytoplasm compared with control cells (Figure 5C). Interestingly, CK treatment exhibited a significant increase in the levels of LC3II protein in all three neuroblastoma cells compared to the untreated control (Figure 5D,E). Beclin-1(BECN) and Atg7 proteins are required for autophagosome formation during autophagy [33]. Treatment with CK significantly enhanced the expression of autophagy marker proteins including BECN, Atg7, and LC3B in SK-N-BE(2) and SH-SY5Y cells (Figure 5F–I). Taken together, these results demonstrate that CK treatment induces early stage autophagy and autophagosome accumulation in neuroblastoma cells.

### 2.6. CK Inhibits Late-Phase Autophagy and the Fusion of Autophagosomes with Autolysosomes

The increase in the amount of cytoplasmic autophagosomes may result from increased synthesis of autophagosomes through upstream processes or the inhibition of lysosomal degradation in later stages. To confirm this possibility, we performed an autophagic flux assay by measuring the total cellular amount of SQSTM1/p62 (sequestosome 1). SQSTM1/p62 is known as an autophagy adapter cargo protein. It is recruited in the autophagosomal membrane and then degraded within lysosomes along with LC3II [33]. Moreover, p62 is also considered a crucial guide of target proteins to the autophagy system for removal from the cell [34]. Thus, downregulation of p62 protein may signify the status of increased autophagic flux [35,36]. In the present study, western blot analysis showed that p62 expression was remarkably increased by dose-dependent CK treatments in SK-N-BE(2) and SH-SY5Y cells (Figure 5F–I). These results indicate that CK treatment induces the accumulation of autophagosomes by inhibiting their degradation in neuroblastoma cells.

Chloroquine (CQ) is a specific late-phase autophagy inhibitor, which inhibits autophagic flux by decreasing autolysosome degradation and, is widely used to inhibit the maturation of autophagosomes into degradative autolysosomes [37,38]. Tandem fluorescence-tagged LC3B (monomeric red fluorescence protein mRFP-GFP-LC3B protein; tfLC3) was used to confirm whether CK treatment can inhibit late-stage autophagy and then cause p62 accumulation. To monitor autophagic flux from autophagosome to autolysosome, GFP and RFP signals showing different pH sensitivity are widely used because GFP signals are quantitatively quenched in acidic compartments, whereas RFP fluorescence is maintained. Therefore, this probe capitalizes on the pH difference between autolysosome (acidic) and autophagosome (neutral), which denotes autophagic flux from autophagosomes (GFP + RFP +; yellow dots) to autolysosomes (GFP - RFP +; red dots).

**Figure 6:**
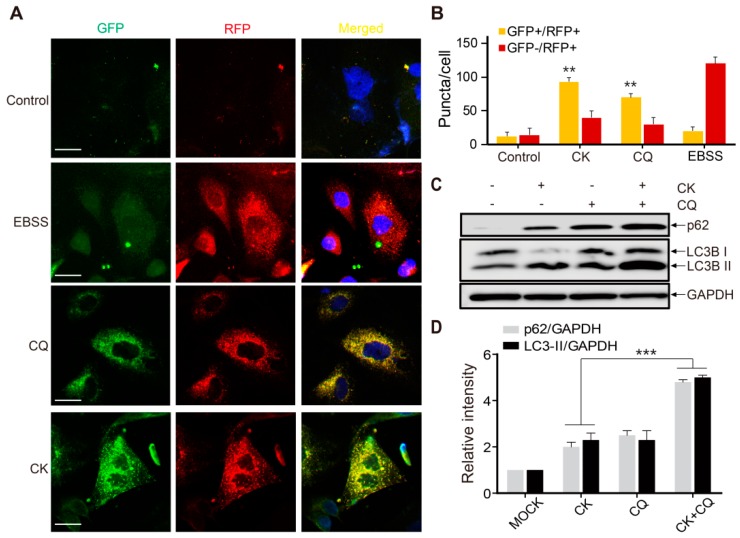
CK inhibits the fusion of autophagosome and autolysosomes. (**A**) mRFP-GFP-LC3B stable SK-N-BE(2) cells were treated with autophagy inhibitor (CQ) and then exposed to CK (5 μM) or starved in EBSS for 24 h. The treated cells were fixed and imaged with fluorescence microscopy. Scale bar: 50 μm. (**B**) Quantitative analysis of double positive (GFP ^+^ /RFP ^+^) and single positive (RFP ^+^) puncta per cell in control, Earle’s balanced salt solution (EBSS)-, CQ-, and CK-treated cells. Data are the mean values of three independent experiments with each count. Values are expressed as the mean ± SD. **: *p* < 0.01 versus control. (**C**) CK increased p62 and LC3B-II expression. SK-N-BE(2) cells were treated with CK for 24 h with or without CQ (10 µM). (**D**) The protein level of LC3B and p62 was analyzed by western blot. Results were normalized to GAPDH. Data are presented as mean ± standard deviation (SD). ***: *p* < 0.001 versus CK-treated cells. CK, Ginsenoside compound K; CQ, chloroquine.

As shown in Figure 6A,B, the number of red puncta was increased in Earle’s balanced salt solution (EBSS)-treated cells (positive control), indicating that autophagy flux was increased. Similar to cells with CQ (10 µM) alone, increased formation of yellow puncta was observed in 5 µM CK-treated SK-N-BE(2) cells without increasing the number of red puncta (Figure 6A,B). Moreover, a CK and CQ combination treatment on SK-N-BE(2) cells further increased the levels of LC3-II and p62 expression in SK-N-BE(2) cells pretreated with CK (5 µM) (Figure 6C,D). Taken together, these results demonstrate that CK blocks fusions between autophagosomes and autolysosomes similar to CQ, indicating that CK can act as a potent autophagy inhibitor.

### 2.7. CK and CQ Combination Therapy Enhances CK-Induced Apoptosis in Human Neuroblastoma Cells

**Figure 7:**
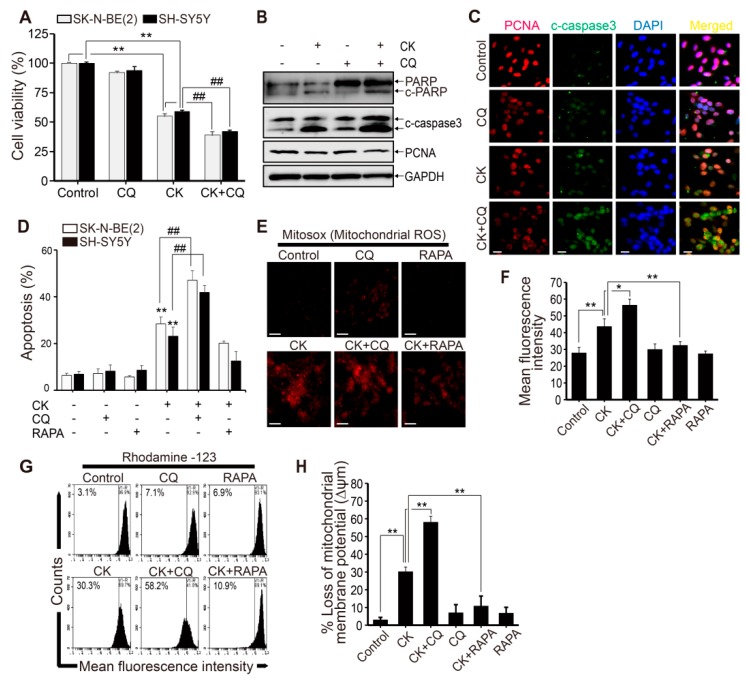
Inhibition of autophagy promotes CK-induced apoptosis via mitochondrial membrane ROS and mitochondrial membrane potential loss in human neuroblastoma cells. (**A**–**D**) Cells were pretreated with 10 μM CQ for 3 h prior to the treatment with CK 5 μM for 24 h. (A) SK-N-BE(2) and SH-SY5Y cell viability was determined by CCK-8 assay after 24 h incubation. (**B**) The protein levels of PARP, cleaved caspase-3, and PCNA in SK-N-BE(2) cells were analyzed by western blot. (**C**) Immunostaining was performed with cleaved caspase-3 and PCNA antibodies in SK-N-BE(2) cells. (**D**) Apoptotic cell death was evaluated by Annexin V/PI analysis with flow cytometry in SK-N-BE(2) and SH-SY5Y cells. Data (**A**) and (**D**) are presented as mean ± SD. **: *p* < 0.01 versus control cells, and ##: *p* < 0.01 versus CK-treated cells. (**E**,**F**) SK-N-BE(2) cells were treated with 5 μM CK with or without CQ (10 µM) or rapamycin (RAPA) (100 nM) for 24 h. (**E**) The MitoSOX™ Red fluorescence intensity was detected by fluorescence microscopy. (F) The corresponding histograms were quantified by Image J. All data are represented as mean ± SD (N = 3) for each group. *: *p* < 0.05, **: *p* < 0.01. (**G**) To determine mitochondrial membrane depolarization, SK-N-BE(2) cells stained with Rhodamine123 dyes were analyzed by flow cytometry. (**H**) Data are presented as mean ± SD (n = 3) for each group. **: *p* < 0.01. CK, Ginsenoside compound K; CQ, chloroquine.

Inhibition of autophagy leads to cancer cell death because autophagy has been proposed as a cancer cell survival mechanism in cancer treatment [16]. To investigate the functional relationship between autophagy and apoptosis after CK treatment, we pretreated human neuroblastoma cells with either CQ (10 µM) or rapamycin (RAPA, 100 nM) before CK treatment (5 μM). As an autophagy inducer, RAPA inhibits mTOR, thus, enhancing autophagy [39]. As shown in Figure 7A, co-treatment of CQ and CK significantly decreased cell viability compared to CK alone in SK-N-BE(2) and SH-SY5Y neuroblastoma cells. Furthermore, western blot results showed that CQ and CK co-treatment increased cleaved caspase-3 and cleaved PARP levels compared with CK alone in SK-N-BE(2) cells (Figure 7B). Consistent with the results of our western blot analysis, immunostaining analysis demonstrated that the CQ and CK combination treatment increased the expression of cleaved caspase-3 higher than CK alone performed in SK-N-BE(2) cells (Figure 7C).

To investigate anti-proliferation effects of the combination treatment with CQ and CK, we examined the expression of proliferating cell nuclear antigen (PCNA), which is a marker of cell proliferation. As a result, we found that the expression levels of PCNA proteins were downregulated to a greater extent with combinatorial treatment with CK and CQ than in CK alone (Figure 7B,C). Next, we examined the rate of cell death by the Annexin/PI method to investigate the effect of CQ and RAPA (100 nM) on CK-induced apoptosis in neuroblastoma cells (Figure 7D). Compared with the control group, CK treatment induced an increase in apoptosis rate, and the co-treatments with CQ further increased apoptosis rate compared to CK alone. However, RAPA treatment did not affect apoptosis rate (Figure 7D). Consequently, the CQ and CK combination treatment not only increased apoptotic cell death but also decreased the survival rate in neuroblastoma cells. Overall, these data strongly suggest that combined treatment with CQ and CK enhances more significant synergistic effects on apoptotic cell death in neuroblastoma than CK treatment alone.

Moreover, to investigate the mechanisms of CK-induced toxicity, mitochondrial ROS levels and mitochondria membrane potential (MMP) (∆ψm) were also measured in SK-N-BE(2) cells. The MitoSOX^TM^ Red reagent was used for detecting superoxide in the mitochondria of CK-induced living cells. MitoSOX fluorescence intensity was clearly increased in CK-treated SK-N-BE(2) cells (Figure 7E,F). The production of mitochondrial ROS, in which CQ and CK coexist, was significantly increased compared with the CK alone group. However, this effect was not found in the group treated with RAPA and CK together (Figure 7E,F). Next, we used Rhodamine 123, a sensitive fluorescent dye, as an indicator of mitochondrial dysfunction to measure MMP (∆ψm) levels (Figure 7G,H). The measured MMP depolarization was significantly accelerated in the combination treatment with CQ in SK-N-BE(2) cells compared to CK alone, but those effects were not found in cells treated with RAPA and CK together (Figure 7G,H). Taken together, these results demonstrate that the CQ and CK combination treatment promotes CK-induced apoptosis through the production of mitochondrial ROS and mitochondrial signaling pathways in neuroblastoma cells.

### 2.8. CQ and CK Combination Treatment Improves the Anti-tumor Effect of CK in Neuroblastoma Xenograft Models

**Figure 8:**
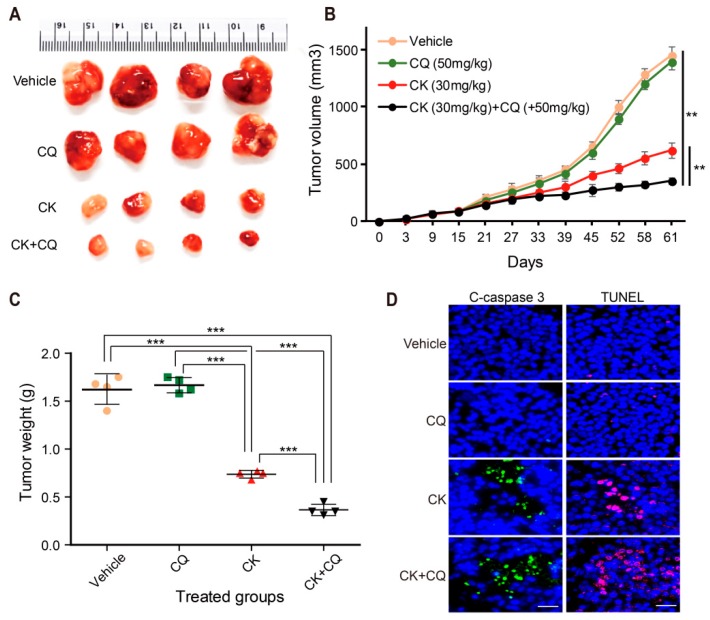
Inhibition of autophagy by CQ improves the CK-induced anti-tumor effect in neuroblastoma xenograft models. (**A**–**C**) Athymic nude mice bearing isogenic SK-N-BE(2) xenograft tumors were treated with vehicle (dimethyl sulfoxide, DMSO), CQ (50 mg/kg), CK (30 mg/kg), and CK in combination with CQ via intraperitoneal injection three times a week. Mice were sacrificed, and tumor samples were collected 61 days after cell injection. (**A**) The picture of different tumor groups. (**B**) Tumor growth curves represented the average values of 5 mice in each group. (**C**) Tumor weight is expressed in a scatter plot. Data (**B**) and (**C**) are presented as mean ± SD. **: *p* < 0.01, ***: *p* < 0.001 versus vehicle (DMSO treatment). (**D**) In each mouse tumor sample group, apoptosis was examined by immunohistochemical staining with cleaved caspase 3 antibody and TUNEL assay. Scale bar, 20 µm.

To investigate the effect of the CQ and CK combination treatment on neuroblastoma cell line in vivo, a subcutaneous xenograft tumor model was established by inoculating SK-N-BE (2) cells into athymic nude mice. As shown in Figure 8A,B, CK treatment (30 mg/kg) significantly inhibited tumor growth in comparison with the vehicle-treated groups, and combination treatment with CK and CQ had a more remarkable anti-tumor effect than CK treatment alone, whereas treatment with only CQ did not show any inhibition of tumor growth. Moreover, the combination treatment also significantly reduced the tumor weight (Figure 8C), but there was no significant body weight change between each animal group (data not shown). The reduction of tumor size in mice treated with CK and combination treatment (CK and CQ) was associated with the increased expression of cleaved caspase 3- as well as TUNEL-positive cells (Figure 8D). Collectively, these data suggest that inhibition of autophagy by CQ may further enhance the anticancer effect of CK in vivo.

## 3. Discussion

In this study, we demonstrate that CK induced apoptosis and ROS generation and inhibited autophagy flux in neuroblastoma cells in vivo and in vitro. Interestingly, chloroquine, an autophagy flux inhibitor, potentiates CK-induced apoptosis. Apoptosis is a systemically well-organized suicide program that removes defective, damaged, or unwanted cells. Autophagy is also an intracellular system that removes cytosolic protein aggregates, damaged organelles, and infectious organisms and contributes to cell survival mechanisms [15,40]. Apoptosis and autophagy are two distinct processes required to maintain homeostasis of cells. In addition, the crosstalk between autophagy and apoptotic cell death was reported in many cancer cell types [10,11,12,13,14]. Hence, we investigated the molecular mechanisms of CK on neuroblastoma cell growth inhibition, cell death, and autophagy to understand the complexity of the relationship between cancer cell apoptosis and autophagy.

Firstly, we found that the growth inhibition by CK in three human neuroblastoma cell lines, such as SK-N-BE(2), SH-SY5Y, and SK-N-SH cells, were dose-dependent (Figure 1B–D), and we also demonstrated that CK induced the accumulation of sub-G1 population (apoptotic cells) and apoptosis in a caspase-dependent manner. We also found that CK significantly inhibited autophagic flux by blocking of autophagosome and lysosome fusion, the step of late-stage autophagy. Similar to our study, CK exerts anticancer effect by triggering apoptosis and autophagy via ROS generation on colon cancer cells [28]. In addition, there is ample evidence that demonstrated that CK has an antitumor effect on various cancers by triggering apoptosis, inhibiting proliferation, or inducing autophagy through multiple pathways [27,41,42,43,44,45]. However, previous studies have limited results such as acting as an autophagy inducer in colon cancer or non-small lung cancer [28,46] or inhibiting cell death by autophagy after brain injury [47]. Thus, the anticancer effect of CK will be controversial whether it contributes to apoptosis or not. In this context, the present study is first to show that CK inhibits autophagy flux in neuroblastoma. It also suggests that that natural product CK can be used for not only inducing apoptosis but also inhibiting autophagic flux in neuroblastoma.

Another cell death type, autophagic cell death (ACD) is an evolutionarily conserved catabolic process that requires the formation of autophagosomes and various autophagy-related proteins, including Atgs, BECN, and LC3B [33,48]. Autophagic flux is defined as a measure of autophagic degradation activity, and LC3-II and p62 are widely used as indicators for the measurement of autophagic flux [49,50,51]. In this study, CK-induced early-stage autophagy in neuroblastoma cells was evidenced by an increased number of autophagosomes and increased expression of the autophagy markers LC3-II, Atg7, and BECN proteins (Figure 5). Additionally, autophagic flux analysis showed that CK treatment induces an increase in p62 expression. Furthermore, CK inhibited autophagic flux by blocking the autophagosome–autolysosome fusion process in neuroblastoma cells, similar to CQ, a late-stage autophagy inhibitor (Figure 6A). According to our results, p62 expression was upregulated by CK treatment in a dose-dependent manner in neuroblastoma cells (Figure 5E,F). Consistent with our data, recent studies demonstrate that upregulation of p62 expression is associated with ACD in breast cancer [52], hepatocellular carcinoma cells [53], and esophageal cancer [54].

The role of autophagy in the treatment of cancer remains still unclear. In many solid tumors, including NB, after chemotherapy, autophagy acts as a survival mechanism of cancer cells, by which it can reduce the effectiveness of chemotherapy [2,55]. Extensive autophagy can lead to cell death, and although autophagy is thought to have a dual role in tumorigenesis, its function is yet to be fully understood [56,57,58,59]. Thus, identification of a clear interaction between autophagy and apoptosis could be an effective approach for cancer treatment. Among the inhibitors of autophagy, chloroquine (CQ), a specific late-phase autophagy inhibitor, inhibits lysosome fusion to the autophagosome and impairs autophagosome maturation into degradative autolysosomes [37,38]. Currently, CQ has been used in cancer clinical trials. Thus, the combination of chloroquine and CK might be a promising strategy in the treatment of neuroblastoma. In the present study, we identified the anti-NB cancer activity of CK through in vitro and in vivo experiments (Figure 1, Figure 2, Figure 3, Figure 4, Figure 7 and Figure 8). Additionally, CK inhibited autophagic flux through blockade of autophagosome and autolysosome fusion in neuroblastoma. This suppression of autophagy with the autophagy inhibitor CQ could significantly enhance CK-induced apoptosis in neuroblastoma cells. More detailed studies found that CK not only stimulated the generation of intracellular ROS, which subsequently triggered mitochondria dysregulation, but also inhibited autophagic flux. However, the relationship between increased ROS level and autophagic flux inhibition by CK remains to be revealed through further studies. This observation has demonstrated the cytoprotective role of autophagy in CK-treated neuroblastoma cells, suggesting that abolition of autophagy can improve the anti-NB cancer effect of CK in vitro. We also noted that the SK-N-BE (2) cell xenograft model confirms the protective effect of autophagy on CK therapy in vivo. As a result, the combination therapy of CK and CQ enhanced the therapeutic effect of CK, which proved that autophagy blocking could further increase the therapeutic effect of NB cells.

Under normal circumstances, ROS formed by the mitochondrial respiration is removed by antioxidant enzymes. However, excessive ROS build-up due to the inhibition of antioxidant activity is toxic to cells [60]. This abnormal ROS production leads to MMP loss, causing leakage of various apoptotic molecules from the mitochondria into the cytoplasm, leading to apoptosis. Thus, ROS is an important mediator of intracellular signaling pathways, including the mitochondrial-mediated apoptosis pathway [60,61]. Recently, a change in ROS level has also been reported to be associated with autophagic death [62,63,64]. Our present results also found that CK-treated neuroblastoma cells have an increased level of intracellular ROS compared to control cells (Figure 3A–C); however, the ROS scavenger NAC significantly reduced ROS production and rescued cell death induced by CK treatment (Figure 3A–D). Interestingly, the CQ and CK combination treatment induced a significant increase in mitochondrial ROS production and loss of MMP compared with CK only treatment, but not RAPA treatment (Figure 6E–H). Additionally, inhibition of autophagy can exacerbate mitochondrial damage and promote mitochondrial-dependent apoptosis in neuroblastoma cells (Figure 6A–D). These results indicate that autophagy can play a protective role against CK-induced apoptosis by eliminating damaged mitochondria. However, further studies on the underlying molecular mechanisms of ROS-mediated apoptosis and autophagy with CK treatment are needed.

One of the advantages in our study is that low concentrations of CK were used against neuroblastoma cells. There are many studies that have reported the inhibitory effect of CK on the growth of various cancer cells, and IC_50_ for HL-60, K562, PC-14, MKN-45, Du145, HCT116, and U373MG were at 11.7, 8.5, 25.9, 56.6, 58.6, 30–50, and 15 μM, respectively [41,42,43,44,45]. In our study, IC_50_ values with CK treatment were 5 μM in SK-N-BE(2) cells, 7 μM in SH-SY5Y cells, and 15 μM in SK-N-SH cells, respectively (Figure 1B–D). It showed a definitely low concentration of CK in neuroblastoma compared to other cell lines, and neuroblastoma cells are much more sensitive to CK than other cancer cells. More importantly, cytotoxicity was not found in normal cells including HUVEC, BJ, and CCD-1079SK cells (Figure 1E–G). This suggests that CK may be a good candidate for NB therapy. Our data provide a preliminary insight into the potential use of CK as an anticancer drug for NB cancer treatment. In addition, further studies are needed to know the mechanism or regulation of cancer-specific effects of CK, and it will be interesting.

**Figure 9:**
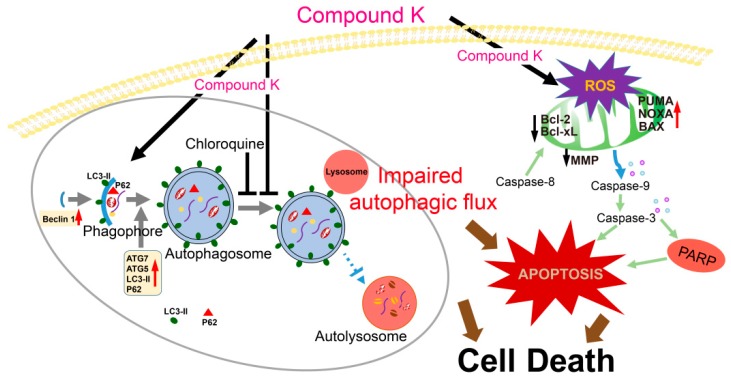
Schematic representation of the proposed mechanisms of CK-induced apoptosis in neuroblastoma cells. CK induced caspase-dependent apoptosis and ROS generation and inhibited autophagy flux. Importantly, ROS scavenger NAC markedly blocked CK-induced ROS generation and apoptosis. Moreover, chloroquine, an autophagy flux inhibitor, augmented the CK-induced apoptosis, mitochondrial ROS generation. Altogether, a combination treatment of CK with chloroquine, a pharmacological inhibitor of autophagy, may be a novel therapeutic potential for the treatment of neuroblastoma.

Overall, we demonstrate that ginsenoside compound K induced ROS generation-mediated apoptosis and inhibited autophagy flux in neuroblastoma cells (Figure 9). Moreover, chloroquine, an autophagy flux inhibitor, potentiates CK-induced apoptosis in vitro and in vivo. To our knowledge, we first report the anticancer effect of CK combination with chloroquine against neuroblastoma cells. The findings suggest that a combination strategy of the ginsenoside CK with chloroquine (autophagy inhibitor) could be a novel therapeutic potential for the treatment of neuroblastoma.

## 4. Materials and Methods

### 4.1. Chemicals and Reagents

CK (purity > 97%) was prepared by a transformation of PPD-type ginsenosides extracted from Korean ginseng using acid-heat treatment. N-acetyl-L-cysteine (NAC), dimethyl sulfoxide (DMSO), 2′,7′dichlorodihydrofluorescein diacetate (DCFH-DA), Carbonyl cyanide m-chlorophenyl hydrazone (CCCP), rapamycin (RAPA), acridine orange (AO), chloroquine (CQ), and an in situ cell death detection kit (TMR red; TUNEL) were purchased from Sigma-Aldrich Chemical Co. (St. Louis, MO, USA). Dulbecco’s modified eagle medium (DMEM), fetal bovine serum (FBS), 0.05% Trypsin-EDTA and penicillin-streptomycin were purchased from Gibco BRL (Grand Island, NY, USA). The following antibodies were used: LC3-I/II was purchased from Sigma; GAPDH, Bcl-2, cleaved caspase-3, caspase-8, PCNA, ATG7, Beclin 1, and p62 (Cell Signaling, Danvers, MA, USA), PARP and Bcl-xL (Santa Cruz Biotechnology, Dallas, TX, USA), Alexa 488/594 conjugated secondary antibodies (Abcam, Cambridge, UK), Annexin V-FITC apoptosis detection kit, propidium iodide (PI), Hoechst 33342 staining kit, and Z-VAD-FMK were purchased from BD Biosciences (SanJose, CA, USA).

### 4.2. Cells and Culture Conditions

Human neuroblastoma (SH-SY5Y and SK-N-BE(2)), human foreskin fibroblasts (CCD-1079SK, and BJ), and human umbilical vein endothelial cell (HUVEC) lines were obtained from the American Type Culture Collection (Manassas, VA, USA). SK-N-SH cells were obtained from the Korean Cell Line Bank (Seoul, Korea). Cells were cultured in DMEM supplement with 10% (v/v) heat-inactivated FBS and 1% (v/v) penicillin-streptomycin in a humidified incubator with 5% CO_2_ atmosphere and 37 °C. CK was prepared in 100% dimethyl sulfoxide, stored in small aliquots at −80 °C and diluted in the cell culture medium as needed.

### 4.3. Cell Viability Assay

Cell viability was assessed using the cell count kit-8 (Dojindo Molecular Technologies, Tokyo, Japan) assay. Cells were seeded in the 96-well plates (1 × 10^4^/well) and exposed to various concentrations of CK (2, 5, 10, 15, 20 µM) for 24 h. Pretreated with NAC (5 mM), CQ (10 µM), RAPA (100 nM), and Z-VAD-FMK (20 µM) for 3 h and then exposed to either with or without 5 µM CK for 24 h. Cells without addition of CK were taken as control. The medium was then removed, and 10 μL of the CCK-8 solution was added to each well of the plate. After 3 h incubation, the absorbance was measured at 450 nm using a microplate reader Synergy TM (BioTek Instruments, Inc., Winooski, Vermont, USA). The cell viability was expressed as the percentage of the control, which was set to 100. All experiments were performed in triplicate and repeated at least three times. The 50% inhibitory concentration (IC_50_) was obtained from the dose-response curve of percent viability (Y) versus tested concentrations (X). IC_50_ was calculated using linear regression analysis in Microsoft Excel.

### 4.4. Colony Formation Assay

For colony formation assay, SK-N-BE(2) and SH-SY5Y cells were placed into a 6-well plate at a density of 1 × 10^3^ cells/well and incubated with different concentrations of compound K at 37 °C for 24 h. The medium was then replaced freshly every day, and cells were grown for 14 days. After staining with 0.5% crystal violet for 30 min, the colonies were visualized and quantified.

### 4.5. Cell Cycle Analysis

SK-N-BE(2) cells were seeded onto 6-well plates at a density of 5 × 10^5^ cells/well and treated with various concentrations of CK for 24 h. Floating and adherent cells were collected with trypsin-EDTA (Sigma-Aldrich) and fixed in 70% ethanol overnight at −20 °C. The cells were washed with cold phosphate buffered saline (PBS) and stained with PI (100 μg/mL) in the dark at 37 °C for 30 min. Then, 10,000 fluorescent events were measured and analyzed with an Accuri C6 flow cytometer (BD Biosciences, San Jose, CA, USA).

### 4.6. Hoechst 33342 Staining

For the Hoechst 33342 staining, SK-N-BE(2) and SH-SY5Y cells were pretreated with 5 mM NAC for 3 h and treated with 5 µM CK for 24 h. The cells were washed with cold PBS and then fixed with cold methanol and stained with Hoechst 33342 (1 μg/mL) for 10 min. The morphology was examined by fluorescence microscopy (CELENA S, Logos Biosystems, Anyang, Korea).

### 4.7. Apoptosis Assay with Annexin V and PI Staining

Apoptotic cell death was determined using apoptosis detection kit (Annexin V-PI: BD Biosciences, San Jose, CA, USA). Briefly, 5 × 10^5^ cells were treated with CK (0, 5, 10, and 20 μM) for 24 h. Cells were harvested and washed once with PBS, and stained with 5 μL of Annexin V-FITC and 5 μL of propidium iodide (PI) for 30 min at 37 °C. The stained cells were analyzed by Accuri C6 flow cytometry. Apoptotic cells (Annexin V-FITC ^+^ /PI^−^ and Annexin V-FITC ^+^ /PI ^+^) were counted and presented as a percentage of the total cell count.

### 4.8. Mitochondrial Membrane Potential (∆ψm) Assay

Cells (2 × 10^5^) were seeded on 6-well plates and treated with either with or without CK for 24 h. Mitochondrial membrane potential changes were monitored by rhodamine 123 using flow cytometry. In brief, cells were incubated with rhodamine 123 (0.1 μg/mL) for 30 min at 37 °C and collected. The fluorescent intensity was measured using flow cytometry. The positive control was treated with CCCP.

### 4.9. Hoechst 33342 and PI Staining

The levels of nuclear condensation and fragmentation were observed by Hoechst 33342 (1 μg/mL) and PI (1 mg/mL). Cells were grown on cover slips and treated with CK at various concentrations (0, 5, 10, and 20 µM) after 24 h. Cells were washed twice with cold phosphate buffered saline (PBS), and fixed in cold methanol for 10 min at room temperature. The fixed cells were washed twice with PBS. The stain solution was dropped on a glass slide and incubated for 20 min at 37 °C, and coverslips were placed on a glass slide prior to the observation by a fluorescence microscopy. Four visual fields were randomly selected from each sample and data were collected from three independent experiments. The cell death rate (%) was expressed as the ratio of PI positive stained cells in total Hoechst stained cell.

### 4.10. RNA Isolation and Reverse Transcription (RT)-PCR

Total RNA was isolated using TRIZOL reagent (Invitrogen, Carlsbad, CA, USA), according to the manufacturer’s protocol, and 5 μg of RNA was used for complementary DNA (cDNA) synthesis using synthesized using Go Script reverse transcription system (Promega, Madison, WI, USA). PCR was carried out with specific primers. The PCR products were electrophoresed on 1.2% agarose gel and visualized on a UV transilluminator using Red Safe (iNtRON Biotechnology, Seongnam, Korea). The relative expression level of a target gene was quantified by normalization with the internal control GAPDH gene. Primers used in the experiment were as follows: (1) human *Bax* (forward) 5′-TCTGACGGCAACTTCAACTG-3′ and (reverse) 5′-TCCCGCCACAAAGATGGTCACG-3′; (2) human *Bcl-2* (forward) 5′-GAGGATTGTGGCCTTCTTTG-3′ and (reverse) 5′-ACAGTTCCACAAAGGCATCC-3′; (3) human *Caspase-3* (forward) 5′-TTTGT TTGTGTGCTTCTGAGCC-3′ and (reverse) 5′-ATTCTGTTGCCACCTTTCGG-3′; (4) human *Caspase-9* (forward) 5′-AACAGGCAAGCAGCAAAGTT-3′ and (reverse) 5′-TCCATCTGTGCCGTAGACAG-3′; (5) human *Puma* (forward) 5′-AGTGTCCTGCGGCCTCTG-3′ and (reverse) 5′-GGAGTCCCATGATGAGATTGT-3′; (6) human *Noxa* (forward) 5′-CGGAGATGCCTGGGAAGAA-3′ and (reverse) 5′-AGGTTCCTGAGCAGAAGAGT-3′; (7) human *GAPDH* (forward) 5′-GAGTCAACGGATTTGGTCGT-3′ and (reverse) 5′-GACAAGCTTCCCGTTCTCAG-3′.

### 4.11. Western Blot Analysis

After treated with various stimuli, cells were lysed for 30 min in radioimmunoprecipitation assay (RIPA) buffer supplemented with protease and phosphatase inhibitor cocktail (Thermo Fisher scientific, Waltham, MA, USA). The samples were heated to 100 °C for 5 min and placed on ice. Whole cell extracts (30 µg protein) were loaded onto a 12% sodium dodecyl sulfate-polyacrylamide gel electrophoresis (SDS-PAGE) gel and transferred onto a polyvinylidene fluoride (PVDF) membrane (Millipore, Burlington, MA, USA). The membrane was blocked with 5% milk or 5% BSA for 1 h at room temperature (RT), and incubated overnight with primary antibodies, followed by horseradish peroxidase (HRP)-conjugated secondary antibodies for 1 h at RT. The blot bands were detected with an enhanced chemiluminescence (GE Healthcare, Piscataway, NJ, USA).

### 4.12. Measurement of Intracellular ROS Generation

The generation of ROS was evaluated by fluorescence microscopy and flow cytometry. ROS production was measured using the dye DCFH-DA (Sigma, MO, USA), as previously described [31,65]. For flow cytometry, cells treated with or without CK for 24 h were rinsed with cold PBS and incubated in 10 μM DCFH-DA for 30 min at 37 °C in the dark. DCF fluorescence was measured using Accuri C6 FACS (BD Biosciences, San Jose, CA, USA), and data was analyzed using Accuri C6 program (BD Biosciences, San Jose, CA, USA). For fluorescence microscopy, cells were seeded on coverslips and treated with or without CK for 24 h. Cells were incubated in 10 μM DCFH-DA for 30 min at 37 °C in the dark, rinsed with cold PBS, and imaged under a fluorescence microscopy. On average, three microscope fields were quantified in three separate cultures per treatment condition. Image J was used to quantify fluorescence intensity. The results were expressed as percentage change from the controls.

### 4.13. Immunofluorescence Staining

Cells were cultured on coverslips and pretreated with 10 μM chloroquine for 3 h prior to treating with 5 μM CK for another 24 h. Cells were then washed twice in PBS, fixed with cold methanol, and permeabilized with 0.2% Triton X-100 in PBS for 20 min. After blocking with 10 % normal goat serum for 1 h, and incubated overnight at 4 °C with primary antibodies (cleaved caspase-3 and PCNA), washed three times in PBS, and incubated in Alexa 488-conjugated anti-rabbit IgG or Alexa-594-conjugated anti-mouse IgG for 1 h. Nuclei were counterstained using Hoechst 33342 (BD Biosciences, San Jose, CA, USA), and the stained cells were observed under a fluorescence microscopy CELENA S. Images were analyzed using ImageJ software version 1.52a (NIH, Bethesda, MD, USA).

### 4.14. Detection of Autophagic Vacuoles by Acridine Orange Staining (AO)

A fluorescent compound, acridine orange (Sigma, St. Louis, MO, USA) is commonly used to measure DNA and RNA levels in cells and can also be used to detect the level of acidic granule compartments within cells undergoing autophagy [66]. Formation of acidic vesicular organelles (AVOs) was quantitated by acridine orange staining [66,67]. SK-N-BE(2) cells (5 × 10^5^/well) were cultured in 24-well culture plates and treated without or with CK (5 μM) for 24 h, then cells were stained with 5 µg/mL acridine orange (Sigma) for 15 min at 37 °C. After washed with PBS, cells were examined under a fluorescence microscope and analyzed by flow cytometry using the Accuri C6 program.

### 4.15. Plasmids Construction

For the generation of enhanced green fluorescent protein-LC3 (EGFP-LC3) transfection vector, the full length of human microtubule-associated protein 1 light chain 3 (LC3; GenBank accession no. NM_022818.5) was amplified by RT-PCR using forward (5′-GTTCTCGAGCTATGCCGTCGGAGAAGA-3′) and reverse (5′-AAGGATCCTTACACTGACAATTTCATCC-3′) primers, and the SK-N-BE(2) cDNA was used as template. The PCR fragment of LC3 protein was digested using with XhoI and BamHI (Enzynomics, Daejeon, Korea) and was cloned into pEGFP-C1 plasmid (Clontech, Palo Alto, CA, USA). All constructs were verified by sequencing. The mRFP-GFP-LC3 expression plasmid was a generous gift from Jaerak Chang (Ajou University, Suwon, Korea).

### 4.16. Cell Transfection and Stable Cell Screening

Transient transfections were performed using Lipofectamine 2000 reagent (Invitrogen, Carlsbad, CA, USA) according to the manufacturer’s protocols. SK-N-BE(2) cells were cultured in DMEM (Gibco) containing 10% fetal bovine serum (FBS) and antibiotics in 5% CO_2_ at 37 °C. When the confluence reached 70% in 6-well culture plates and then transfected with 5 μg plasmid per well (plasmid (μg): Lipofectamine 2000 (μL) = 1:3). Forty-eight hours after transfection, cells were digested with 0.25% trypsin and the cultures were transferred to the plates for further culture with complete DMEM containing 1000 mg/L G418 for 14 days. When the amount of resistant cell clones was observed, they were digested with 0.25% trypsin and then transferred to a new culture flask using an aseptic pipette for further culture. Selected cells were used for additional autophagy analysis.

### 4.17. Detection of GFP-LC3 or mRFP-GFP-LC3B Assay

After the pEGFP-LC3 or mRFP-GFP-LC3B stable SK-N-BE(2) cells were treated with CK or/and CQ for 24 h, the cells were fixed, and mounted. Images from slides were observed under a fluorescence microscope CELENA S.

### 4.18. Measurement of Mitochondrial ROS Generation

The formation of mitochondrial ROS was measured using MitoSOX^TM^ Red (Life technologies, Eugene, OR, USA) assay. MitoSOX^TM^ Red reagent is oxidized by superoxide once inside the mitochondria, and is converted to a fluorogenic oxidation product upon binding to nucleic acids [68]. For microscopy, cells were treated with 5 μM CK for 24 h in the presence or absence of CQ and RAPA. After staining with MitoSOX^TM^ Red, images were collected using a fluorescence microscopy. Mean fluorescence intensity of images was analyzed using the Image J.

### 4.19. Tumor Xenograft Studies

Animal studies were performed as described previously [69]. The mice were maintained in a specific pathogen free environment. SK-N-BE(2) cells (3 × 10^7^) in 100 µL (PBS: Matrigel = 1:1) were injected subcutaneously into the right flank of each mouse. A week after, tumor injected mice were randomly divided into vehicle, CK, CQ, and CK + CQ groups (n = 5 per group). The vehicle group was given DMSO, and the treatment groups were injected with CK (30 mg/kg), CQ (50 mg/kg) (Sigma-Aldrich) or both drugs, respectively. All drug injection was administered intraperitoneal three times per week. Body weight and tumor volume were also measured three times a week, and the tumor volume was calculated using the following formula: tumor size (mm^3^) = π/6 × (length × width × height). Tumor inoculated mice were sacrificed after 60 days. All experimental procedures were approved by the Institutional Animal Care and Use Committee of Chonbuk National University (permit number: CBNU-2018-013).

### 4.20. Histology and TdT-Mediated dUTP Nick end Labelling (TUNEL)

All immunohistochemistry experiments were performed as described previously [69]. Paraffin-embedded samples were sliced into 4 µm sections. After antigen retrieval, tissue samples were blocked using blocking buffer (5% goat serum) for 30 min. After washed three times with PBS, samples were incubated in primary antibody (cleaved caspase 3, 1:250) at 4 °C overnight. After washed three times with PBS, samples were incubated for 1 h at RT with an Alexa-488 conjugated antibody. TdT-mediated dUTP nick end labelling (TUNEL) was performed with an In Situ Cell Death Detection (TMR red) Kit (Sigma; St. Louis, MO, USA) according to the manufactured instructions.

### 4.21. Statistical Analysis

All experiments were performed in triplicate and data were expressed as mean ± standard deviation (SD). Statistical analysis was performed using SPSS (version 12.0, SPSS Inc., Chicago, IL, USA). Significant differences between treatment effects were determined by one-way analysis of variance or two-tailed Student’s t-test analysis, and *p* < 0.05 (*), *p* < 0.01 (**), and *p* < 0.001 (***) were considered statistically significant.

## Abbreviations

CK	Compound K
ROS	Reactive Oxygen Species
PPD	ProtoPanaxaDiol
AO	Acridine Orange
PCNA	Proliferating Cell Nuclear Antigen
CCK-8	Cell Count Kit-8
CQ	Chloroquine
RAPA	Rapamycin
AVOs	Acidic Vesicle Organelles
MMP	Mitochondria Membrane Potential
